# RNA Deep Sequencing Reveals Novel Candidate Genes and Polymorphisms in Boar Testis and Liver Tissues with Divergent Androstenone Levels

**DOI:** 10.1371/journal.pone.0063259

**Published:** 2013-05-16

**Authors:** Asep Gunawan, Sudeep Sahadevan, Christiane Neuhoff, Christine Große-Brinkhaus, Ahmed Gad, Luc Frieden, Dawit Tesfaye, Ernst Tholen, Christian Looft, Muhammad Jasim Uddin, Karl Schellander, Mehmet Ulas Cinar

**Affiliations:** 1 Institute of Animal Science, University of Bonn, Bonn, Germany; 2 Department of Animal Production and Technology, Faculty of Animal Science, Bogor Agricultural University, Bogor, Indonesia; 3 Department of Animal Production, Faculty of Agricultural, Cairo University, Giza, Egypt; 4 Department of Medicine, Faculty of Veterinary Science, Bangladesh Agricultural University, Mymensingh, Bangladesh; Wageningen UR Livestock Research, Netherlands

## Abstract

Boar taint is an unpleasant smell and taste of pork meat derived from some entire male pigs. The main causes of boar taint are the two compounds androstenone (5α-androst-16-en-3-one) and skatole (3-methylindole). It is crucial to understand the genetic mechanism of boar taint to select pigs for lower androstenone levels and thus reduce boar taint. The aim of the present study was to investigate transcriptome differences in boar testis and liver tissues with divergent androstenone levels using RNA deep sequencing (RNA-Seq). The total number of reads produced for each testis and liver sample ranged from 13,221,550 to 33,206,723 and 12,755,487 to 46,050,468, respectively. In testis samples 46 genes were differentially regulated whereas 25 genes showed differential expression in the liver. The fold change values ranged from −4.68 to 2.90 in testis samples and −2.86 to 3.89 in liver samples. Differentially regulated genes in high androstenone testis and liver samples were enriched in metabolic processes such as lipid metabolism, small molecule biochemistry and molecular transport. This study provides evidence for transcriptome profile and gene polymorphisms of boars with divergent androstenone level using RNA-Seq technology. Digital gene expression analysis identified candidate genes in flavin monooxygenease family, cytochrome P450 family and hydroxysteroid dehydrogenase family. Moreover, polymorphism and association analysis revealed mutation in *IRG6, MX1, IFIT2, CYP7A1, FMO5* and *KRT18* genes could be potential candidate markers for androstenone levels in boars. Further studies are required for proving the role of candidate genes to be used in genomic selection against boar taint in pig breeding programs.

## Introduction

Boar taint is an off-odor and off-flavor meat trait, mainly caused by high levels of androstenone, skatole and/or indole in adipose tissue [1]. The taint has been described as being similar to urine and manure and may occur in meat from uncastrated sexually mature male pigs [2]. Consumers commonly show a strong aversion to tainted meat. Currently, surgical castration of male piglets is a common practice in many countries to produce taint-free porcine meat [3]. However, castration is undesirable due to ethical and economical concerns [4] and rearing entire males instead of castrates has a number of advantages including higher efficiency, leaner carcasses and lower faecal and urinary nitrogen losses [5]. By 2018, castration of piglets is going to be banned in the European Community [6]. Consequently, there is an urgent need to develop alternative methods to prevent tainted meat. In literature, it has been mentioned that lowering the slaughter weight or choosing a definite breed can reduce the boar taint [7], however, these could lead to some economical drawbacks. Skatole is a derivative of tryptophan produced in the hindgut of pigs by intestinal bacteria. The level of intestinal skatole production is mainly dependent on nutritional factors and no genetic control has been demonstrated so far [8]. On the other hand, for androstenone high heritability estimates (*h^2^* = 0.25 to 0.87) and differences between sire lines have been reported[9]; [10]; [11]. Consequently molecular breeding seems to be a promising way to produce pigs without boar taint.

Androstenone is synthesized in the testis from pregnenolone [8]; [12]; [13], in relation with sexual development. It is mainly degraded in liver and deposited in adipose tissue because of its lipophilic properties [14]. Metabolism of androstenone is presented in two phases: phase I consists metabolism by hydrogenation and phase II consists metabolism by sulfoconjugation in testis or in liver [8]; [14]; [15]; [16]. Therefore, in theory, high levels of androstenone in fat can be dedicated to a high intensity of testicular synthesis and/or a low intensity of liver degradation [8]. This phenomenon is mainly controlled by enzymes and regulatory proteins such as cytochrome P450 and hydroxysteroid sulfotransferase family. Cytochrome P450s (CYPs) act as mono-oxygenases, with functions ranging from the synthesis to the degradation of endogenous steroid hormones [17]. Androstenone synthesis is initiated by cleavage of cholesterol to produce pregnenolone. This reaction is catalysed by the enzyme *CYP11A* [8]. Formation of 16-androstene steroids from pregnenolone is orchestrated by *CYB5* which causes overproduction of 16-androstene steroids in testis [18]; [19]. Two other cytochrome P450 enzymes *CYP17* and *CYP21* have also been investigated for the involvement in steroidogenesis [8]. 3-β-hydroxysteroid dehydrogenase (3β-HSD) enzyme encoded by *HSD3B* gene [20] reduces androstenone to β-androstenol in pig liver microsomes [14]. The 16-androstene steroids in the liver and testis are sulfoconjugated by hydroxysteroid sulfotransferase (*SULT2A*) [16]; [21].

A number of quantitative trait loci (QTL) and genome-wide association analysis have been conducted for androstenone in the purebred and crossbred pig populations [2]; [22]; [23]; [24]; [25]; [26]. Gene expression analysis has been used to identify candidate genes related to the trait of interest. Several candidate genes have been proposed for divergent androstenone levels in different pig populations by global transcriptome analysis in boar testis and liver samples [27]; [28]; [29]. Functional genomics provides an insight into the molecular processes underlying phenotypic differences [30] such as androstenone levels. RNA-Seq is a recently developed next generation sequencing technology for transcriptome profiling that boosts identification of novel and low abundant transcripts [31]. RNA-Seq also provides evidence for identification of splicing events, polymorphisms, and different family isoforms of transcripts [32]. The major aim of this study was to elucidate the genes involved in androstenone metabolism in testis and liver tissues using RNA-Seq technology. For this purpose, we analyzed differential expression of genes between high and low androstenone sample groups and polymorphisms that appear on the differentially expressed genes.

## Results

### Analysis of RNA-Seq Data

We sequenced cDNA libraries from 10 samples per tissue using Illumina HiSeq 2000. The sequencing produced clusters of sequence reads with maximum 100 base-pair (bp) length. After quality filtering the total number of reads for testis and liver samples ranged from 13.2 million (M) to 33.2 M and 12.1 M to 46.0 M, respectively. There was no significant difference in the number of reads from low and high androstenone samples (*p* = 0.68). Total number of reads for each tissue group and the number of reads mapped to reference sequences are shown in Table 1 and 2. In case of testis 42.20% to 50.34% of total reads were aligned to reference sequence whereas, in case of liver 40.8% to 56.63% were aligned.

**Table 1:** Summary of sequence read alignments to reference genome in testis samples.

Group	Sample	Total number of reads	Un-mapped reads	Mapped reads	Percentage of unmapped reads	Percentage of mapped reads
Low androstenone	A1	15,142,756	7,811,096	7,331,660	51.50	48.50
	A2	13,221,550	6,564,679	6,656,871	49.66	50.34
	A3	32,389,084	16,697,785	15,691,299	51.50	48.50
	A4	27,068,779	14,123,318	12,945,461	52.10	47.90
	A5	27,015,712	14,465,669	12,550,043	53.54	46.46
High androstenone	A6	32,691,057	18,919,738	13,771,319	57.80	42.20
	A7	33,206,723	17,271,473	15,935,250	51.20	48.80
	A8	15,111,453	7,764,418	7,347,035	51.38	48.62
	A9	14,330,069	8,070,092	6,259,977	56.31	43.69
	A10	15,605,400	8,276,052	7,329,348	53.30	46.70

**Table 2:** Summary of sequence read alignments to reference genome in liver samples.

Group	Sample	Total number of reads	Un-mapped reads	Mapped reads	Percentage of unmapped reads	Percentage of mapped reads
Low androstenone	B1	29,549,267	15,632,809	13,916,458	53.50	46.50
	B2	46,050,468	25,270,695	20,779,773	54.87	45.13
	B3	16,420,055	7,659,515	8,760,540	46.64	53.36
	B4	13,323,763	6,989,584	6,334,179	52.46	47.54
	B5	27,085,837	11,747,225	15,338,612	43.37	56.63
High androstenone	B6	28,976,693	16,123,777	12,852,916	55.64	44.36
	B7	12,755,487	5,879,896	6,875,591	46.10	53.90
	B8	45,203,089	18,443,608	26,759,481	59.20	40.8
	B9	14,559,329	8,540,379	6,018,950	58.66	41.34
	B10	14,527,329	8,062,992	6,464,337	55.51	44.49

### Differential Gene Expression Analysis

**Figure 1:**
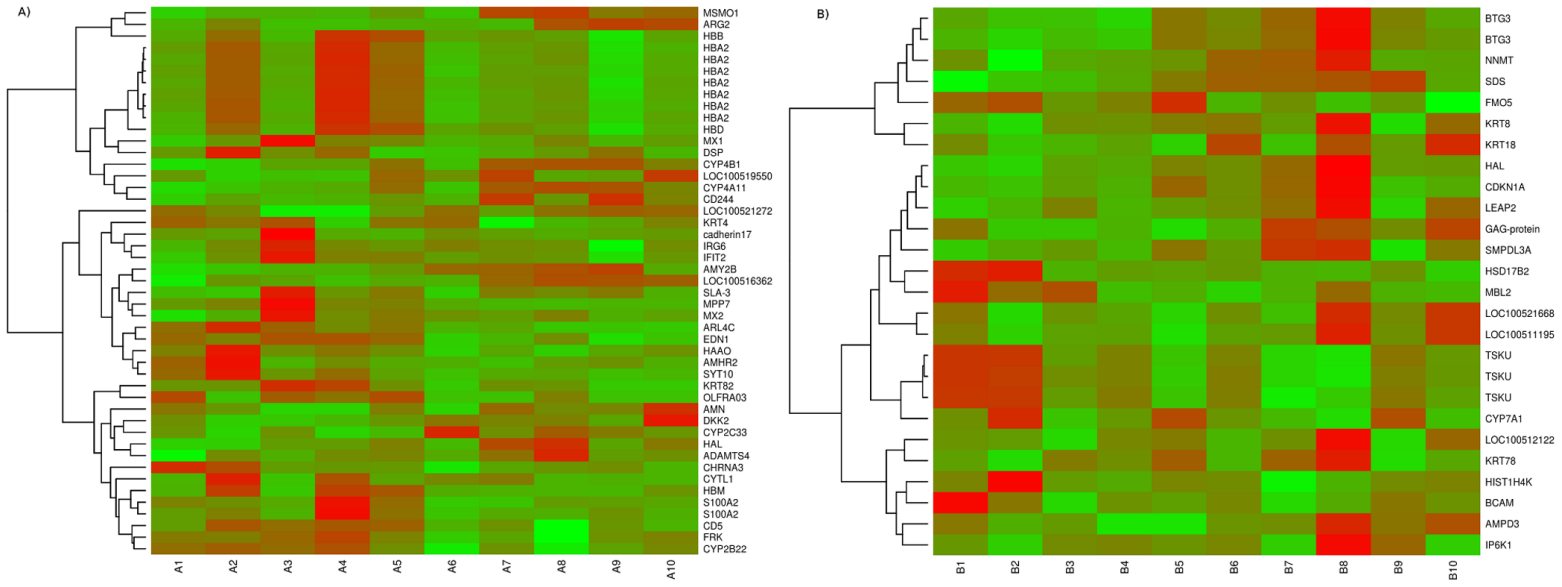
Heatmap showing differentially expressed genes in (A) testis and (B) liver samples. The red blocks represent over expressed genes, and the green blocks represent under expressed genes. Legend: A1–A5 testis with low androstenone and A6–A10 testis with high androstenone, B1–B5 liver with low androstenone and B6–B10 liver with high androstenone.

Differential gene expression for testis and liver with divergent androstenone levels were calculated from the raw reads using the R package DESeq [33]. The significance scores were corrected for multiple testing using Benjamini-Hochberg correction. We used a negative binomial distribution based method implemented in DESeq to identify differentially expressed genes (DEGs) in testis and liver with divergent androstenone levels. The smear plots for differential expression between high and low androstenone levels in testis and liver are given in Figure S1. A GLM analysis (implemented in DESeq package) was also done on the same data set to identify genes with a significant difference between within group deviance and between group deviances. Finally DEGs were selected based on criteria *p*_adjusted_ <0.05 and fold change ≥1.5 from first analysis and *p*_adjusted_ <0.05 in GLM analysis (Table S1). A total of 46 and 25 DEGs were selected from the differential expression analysis for testis and liver tissues respectively (Table 3 and 4). In testis tissues, 14 genes were found to be highly expressed in high androstenone group whereas, 32 genes were found to be highly expressed in low androstenone group. In the liver tissue, 9 genes were found to be highly expressed in high androstenone group whereas, 16 genes were found to be highly expressed in low androstenone group (Table 3 and 4). The range of log fold change values for DEGs was from −4.68 to 2.90 for testis and from −2.86 to 3.89 for liver. Heat maps (Figures 1A and 1B) illustrate the DEGs identified in high and low androstenone testis and liver tissues.

**Table 3:** Differentially expressed genes in testis androstenone samples.

Gene	Orthologue gene description	Reference ID	Log fold change	*p*-adj.
DKK2	Dickkopf homolog 2	XM_003129269.1	2.89	4.46e-06
AMN	Amnionless homolog	XM_001925648.2	2.28	0.025
LOC100519550	LOC100519550	XM_003127761.1	2.22	9.67e-12
CYP4B1	Cytochrome P450 family 4 subfamily B. polypeptide 1	XM_003128017.1	2.20	8.55e-10
CD244	CD244 molecule natural killer cell receptor 2 B4	XM_001928325.2	2.15	5.35e-08
ADAMTS4	A disintegrin and metalloproteinase with thrombospondinmotifs 4-like	XM_001927507.2	2.10	0.04
CYP4A11	Cytochrome P450 family 4 subfamily A polypeptide 11	XM_003128032.1	2.03	3.38e-06
HAL	Histidine ammonia-lyase	XM_001925061.1	2.03	0.014
CYP2C33	Cytochrome P450 2C33	NM_214414.1	1.91	0.04
AMY2B	Amylase, alpha 2B (pancreatic)	XM_003125887.1	1.85	0.002
ARG2	Arginase type II	XM_001928679.2	1.84	6.96e-06
LOC100516362	LOC100516362	XM_003124870.1	1.74	0.022
LOC100521272	LOC100521272	XM_003126855.1	1.71	4.46e-05
MSMO1	Methylsterol monooxygenasse 1	NM_213752.1	1.66	5.55e-08
KRT4	Keratin 4	XM_001927218.2	−1.52	0.02
MPP7	Membrane protein, palmitoylated 7	XM_003130762.1	−1.54	0.0004
DSP	Desmoplakin	XM_003128168.1	−1.55	6.03e-05
AMHR2	Anti-Mullerian hormone receptor, type II	XM_003126187.1	−1.58	0.025
SLA-3	MHC class I antigen 3	AB105388.1	−1.60	5.15e-07
HAAO	3-hydroxyanthranilate 3,4-dioxygenase	XM_003125193.1	−1.61	0.004
MX1	Myxovirus (influenza virus) resistance 1	NM_214061.1	−1.62	1.63e-09
MX2	Myxovirus (influenza virus) resistance 2	NM_001097416.1	−1.63	7.47e-06
IFIT2	Interferon-induced protein with tetratricopeptide repeats 2	XM_001928671.2	−1.64	0.0094
HBB	Hemoglobin, beta	NM_001144841.1	−1.69	2.12e-08
ARL4C	ADP-ribosylation factor-like 4C	XM_003133753.1	−1.72	0.04
EDN1	Endothelin 1	NM_213882.1	−1.73	0.004
HBM	Hemoglobin, mu	XM_003124683.1	−1.74	0.04
HBD	Hemoglobin, delta	XM_003129515.1	−1.83	1.93e-07
HBA2	Hemoglobin, Alpha 2	XM_003124688.1	−1.90	7.42e-11
HBA2	Hemoglobin, Alpha 2	XM_003124690.1	−1.90	2.17e-10
HBA2	Hemoglobin, Alpha 2	XM_003124687.1	−1.93	1.87e-11
HBA2	Hemoglobin, Alpha 2	XM_003124689.1	−1.95	2.62e-11
HBA2	Hemoglobin, Alpha 2	XM_003124685.1	−1.97	1.87e-11
HBA2	Hemoglobin, Alpha 2	XM_003124684.1	−1.97	1.87e-11
HBA2	Hemoglobin, Alpha 2	XM_003124686.1	−1.99	1.38e-11
FRK	Fyn-related kinase	XM_001925792.2	−2.12	0.002
IRG6	Inflammatory response protein 6	NM_213817.1	−2.17	7.24e-07
SYT10	Synaptotagmin 10	XM_001927016.2	−2.23	9.03e-05
S100A2	S100 calcium binding protein A2	XM_001929559.1	−2.35	0.0008
CD5	CD5 molecule	XM_003122679.1	−2.42	0.02
CYP2B22	Cytochrome P450 2B22	NM_214413.1	−2.48	0.02
CYTL1	Cytokine-like 1	XM_003128849.1	2.82	0.002
S100A2	S100 calcium binding protein A2	XM_001929556.1	−2.83	1.42e-07
CHRNA3	Cholinergic receptor, nicotinic, alpha 3	XM_001925760.2	−3.45	5.07e-08
OLFRA03	Olfactory receptor 3A1	XM_001926523.1	−4.12	0.01
KRT82	Keratin 82	XM_003126157.1	−4.68	1.49e-09

**Table 4:** Differentially expressed genes in liver androstenone samples.

Gene	Orthologue gene description	Reference ID	Log fold change	*p*-adj.
LOC100512122	LOC100512122	XM_003130359.1	3.89	1.10e-14
LOC100511195	LOC100511195	XR_115925.1	3.57	9.26e-15
IP6K1	Inositol hexakisphosphate kinase 1	XM_001925759.2	3.04	0.002
AMPD3	Adenosine monophosphate deaminase 3	XM_003135226.1	2.99	0.0004
LOC100521668	LOC100521668	XR_116002.1	2.52	7.77e-08
SDS	Serine dehydratase	XM_001928302.2	2.12	8.15e-05
BTG3	BTG family member 3	XM_003132741.1	2.12	1.51e-06
KRT78	Keratin 78	XM_001927194.2	2.09	7.80e-05
SMPDL3A	Sphingomyelin phosphodiesterase, acid-like 3A	XM_003121227.1	1.99	9.38e-05
KRT8	Keratin 8	NM_001159615.1	1.96	6.50e-05
LEAP2	Liver expressed antimicrobial peptide 2	NM_213788.1	1.94	3.84e-06
HAL	Histidine ammonia-lyase	XM_001925061.1	1.91	3.36e-06
NNMT	Nicotinamide N-methyltransferase	NM_001123146.1	1.86	3.83e-05
BTG3	BTG family member 3	NM_001097517.1	1.70	0.0007
KRT18	Keratin 18	XM_003126180.1	1.69	0.010
CDKN1A	Cyclin-dependent kinase inhibitor 1A	XM_001929558.1	1.67	6.50e-05
TSKU	Tsukushi small leucine rich proteoglycan homolog	XM_003129674.1	−1.72	0.0017
FMO5	Flavin containing monooxygenase 5	XM_001928594.1	−1.75	0.0043
TSKU	Tsukushi small leucine rich proteoglycan homolog	XM_003129672.1	−1.81	0.002
TSKU	Tsukushi small leucine rich proteoglycan homolog	XM_003129673.1	−1.81	0.002
CYP7A1	Cytochrome P450, family 7, subfamily A, polypeptide 1	NM_001005352.2	−1.87	3.32e-07
HIST1H4K	Histone cluster 1, H4k	XM_001928022.2	−2.60	0.001
MBL2	Mannose-binding lectin (protein C) 2	NM_214125.1	−2.79	0.0001
BCAM	Basal cell adhesion molecule	XM_003127227.1	−2.83	0.017
HSD17B2	Hydroxysteroid (17-beta) dehydrogenase 2	NM_001167649.1	−2.86	3.92e-09

### Biological Function Analysis for DEGs

**Figure 2:**
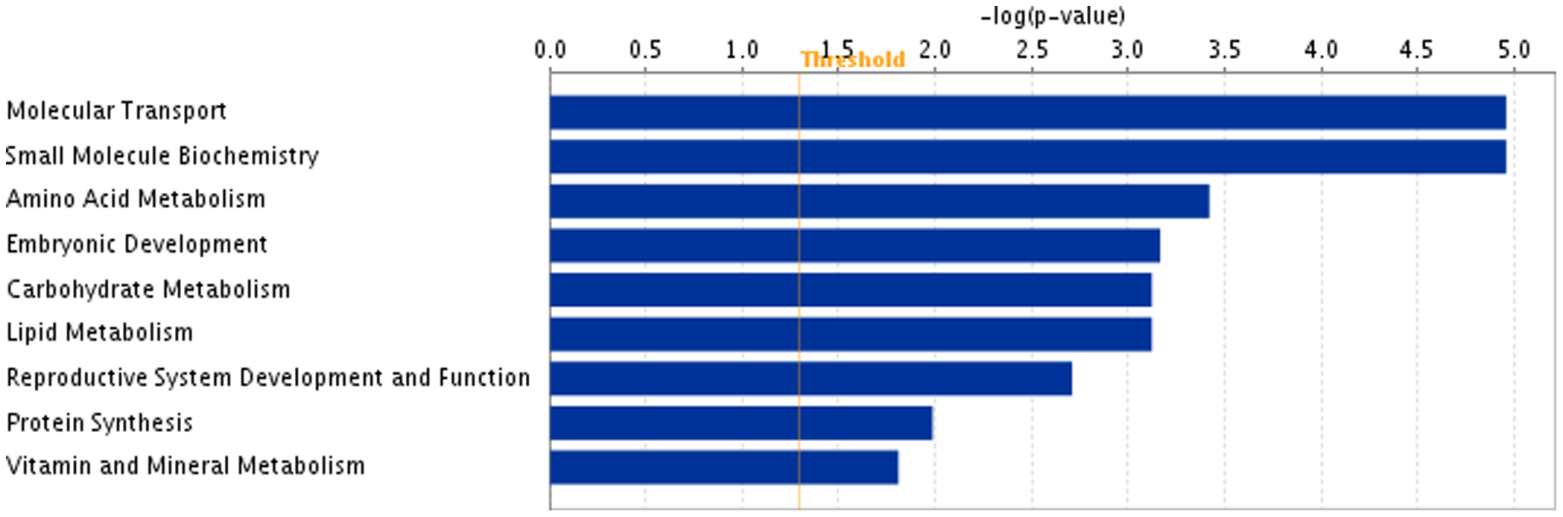
Functional grouping of DEGs in testis with high and low androstenone using Ingenuity Pathways Analysis (IPA) software. The most significant functional groups (*p*<0.05) are presented graphically. The bars represent the *p*-value on a logarithmic scale for each functional group.

To investigate gene functions and to reveal the common processes and pathways among the selected DEGs, Ingenuity Pathway Analysis (IPA) software (Ingenuity Systems, www.ingenuity.com) was used. In testis samples, out of 46 DEGs 39 were assigned to a specific functional group based on the information from IPA (Figure 2). A large proportion (84.7%) of the DEGs from testis high androstenone group fell into Gene Ontology (GO) categories such as molecular transport, small molecule biochemistry, amino acid metabolism, embryonic development, carbohydrate metabolism, lipid metabolism and reproductive system development and function (Figure 2). The genes classified into each functional group are listed in the Table 5.

**Table 5:** Functional categories and corresponding DEGs in high androstenone testis tissues.

Function	Number of genes	*p*-value*	Genes
Molecular transport	9	1.00E-05 to 4.96E-02	*HBB, HBD, HBA1/HBA2, CYP4A11, EDN1, MARCO, AMN, CD44, CD5*
Small molecule biochemistry	12	1.00E-05 to 4.95E-02	*HBB, HBD, ARG2, HBA1/HBA2,CYP4B1, MX1, CYTL1, CYP4A11, MARCO, MSMO1, DSP*
Amino acid metabolism	4	3.80E-04 to 3.48E-02	*ARG2, EDN1, HAL, FRK*
Embryonic development	4	6.80E-04 to 4.40E-02	*HBB, HBD, CYTL1, EDN1*
Carbohydrate metabolism	3	7.54E-04 to 4.96E-02	*CD244, EDN, CYTL1*
Lipid metabolism	7	7.54E-04 to 4.96E-02	*CD244, EDN1, CYP4A11, HBB, MARCO, MSMO1, DSP*
Reproductive system development and function	2	1.95E-03 to 4.96E-02	*NQO1, TNC*
Protein synthesis	3	1.03E-02 to 2.70E-02	*HBA1/HBA2, HBB, ADAMTS4*
Energy production	2	1.64E-03 to 2.43E-02	*EDN1, MARCO*
Vitamin and mineral metabolism	3	1.50E-02 to 2.37E-02	*EDN1, CD244, CD5*

* Numbers in the *p*-value column showed a range of *p*-values for the genes from each category.

**Figure 3:**
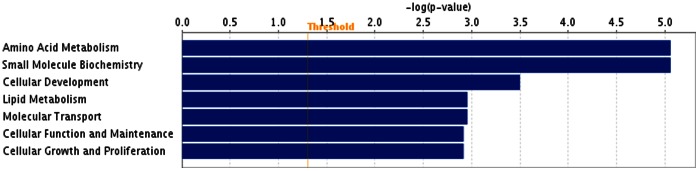
Functional grouping of DEGs in liver with high and low androstenone using Ingenuity Pathways Analysis software. The most significant functional groups (*p*<0.05) are presented graphically. The bars represent the *p*-value on a logarithmic scale for each functional group.

For the liver androstenone samples, out of 25 DEGs, 22 could be assigned to a specific functional group based on the information from IPA (Figure 3). A large proportion (88.0%) of the DEGs from liver high androstenone group was enriched with GO functional categories such as amino acid metabolism, small molecule biochemistry, cellular development, lipid metabolism, molecular transport, cellular function and maintenance and cellular growth and proliferation (Figure 3). The genes classified into each functional group are listed in Table 6.

**Table 6:** Functional categories and corresponding DEGs in high androstenone liver tissues.

Function	Number of genes	*p*-value*	Genes
Amino acid metabolism	3	8.71E-06 to 3.49E-02	*HAL, SDS, CDKN1A*
Small molecule biochemistry	8	8.71E-06 to 2.51E-02	*HAL, CYP7A1, MBL2, AMPD3, HSD17B2, IP6K1, SDS, CDKN1A*
Cellular development	4	3.15E-04 to 2.49E-02	*CDKN1A, KRT8, HIST1H4A, MBL2*
Lipid metabolism	5	1.10E-03 to 2.41E-02	*CYP7A1, MBL2, HSD17B2, IP6K1, CDKN1A, KRT8*
Molecular transport	3	1.11E-03 to 4.41E-02	*CYP7A1, MBL2, CDKN1A*
Cell function and maintenance	4	1.20E-03 to 4.90E-02	*CDKN1A, MBL2, KRT8, KRT18*
Cell growth and proliferation	3	1.20E-03 to 2.90E-02	*CDKN1A, MBL2, KRT8*

* Numbers in the *p*-value column showed a range of *p*-values for the genes from each category.

### Validation of Selected DEGs with Quantitative Real Time PCR (qRT-PCR)

**Figure 4:**
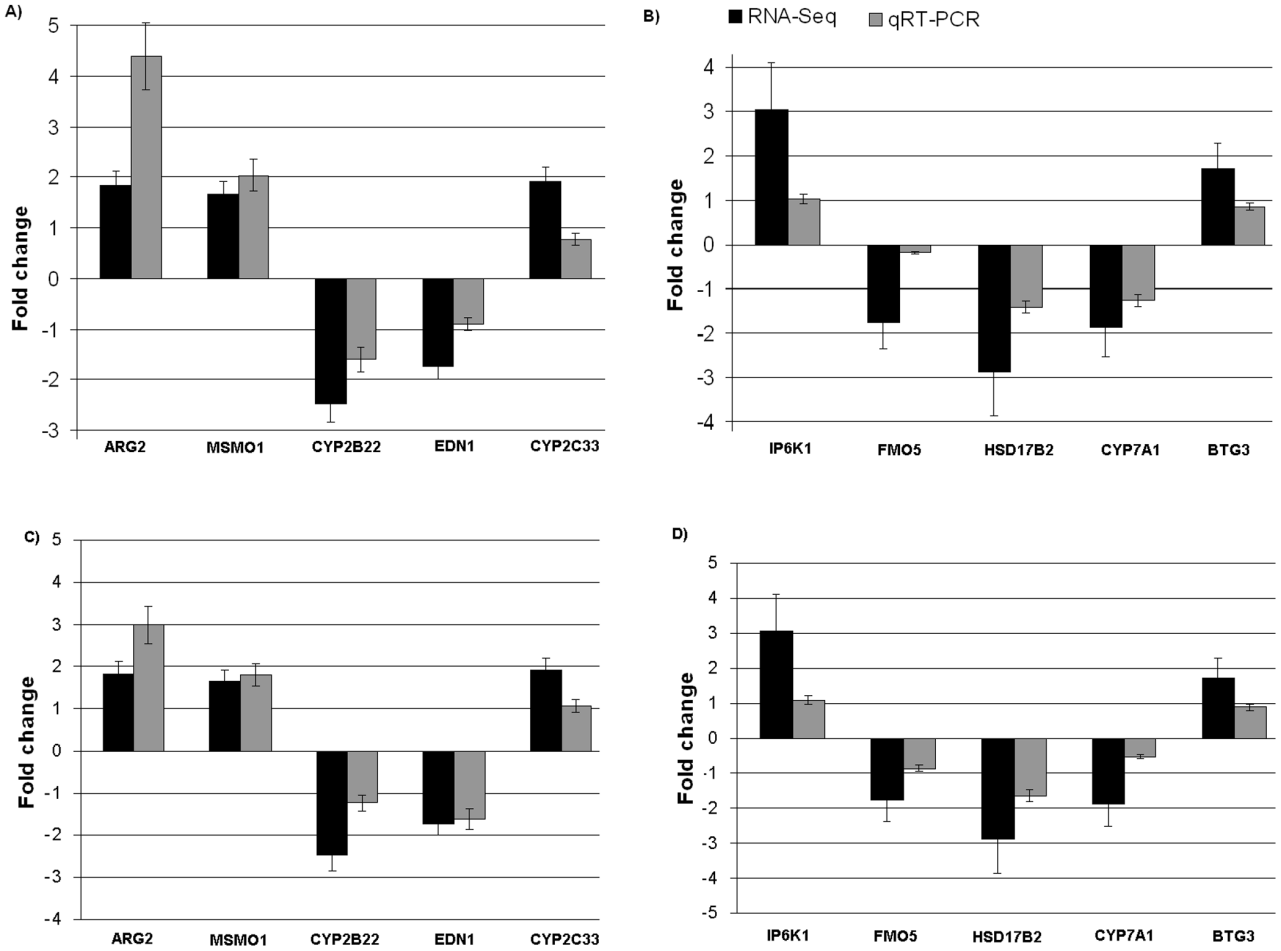
qRT-PCR validations for ten DEGs from divergent androstenone levels in (A and C) testis and (B and D) liver samples. Fold change determined via division of high androstenone group gene expression value by low androstenone group gene expression value.

In order to validate the RNA-Seq results, on the basis of differential expressions and functions related to androstenone, a total of 10 genes were selected and quantified using qRT-PCR. *ARG2, CYP2C33*, *MSMO1*, *EDN1* and *CYP2B22* genes from testis samples and *IP6K1, BTG3*, *CYP7A1*, *FMO5* and *HSD17B2* genes from liver samples were selected. For this purpose, the same samples used in the deep sequencing were used. Comparison of qRT-PCR data for 10 selected genes showed complete concordance of expression with the RNA-Seq results (Figure 4A and 4B). To further validate the expression of selected genes more robustly, new grouping of independently high (n = 5) and low (n = 5) androstenone are done among the remaining 90 pigs. The mRNA expressions of selected genes showed similar pattern of expression in this new groups (Figure 4C and D). Gene expression values for qRT-PCR were normalized using housekeeping genes *PPIA* and *GAPDH* [34].

### Gene Variation Analysis

In total 222,225 and 202,249 potential polymorphism were identified in high and low androstenone testis groups. Among these identified polymorphisms, 8,818 in high androstenone group and 8,621 in low androstenone group were global polymorphisms with reference and accession identifiers in dbSNP database. Similarly in liver high and low androstenone samples 169,181 and 164,417 potential polymorphisms were identified. There were 6,851 global polymorphisms in high androstenone liver sample and 6,436 global polymorphisms in low androstenone liver sample.

Polymorphisms identified in DEGs for testis and liver samples are given in Table 7 and Table 8. In the testis samples 12 gene polymorphisms were identified in 8 DEGs (Table 7). Additionally our results of deep sequencing in limited number of animals revealed that mutations for the genes *CD244* and *ARG2* were specific for high androstenone testis tissues, whereas mutations in genes *IFIT2, DSP* and *IRG6* were specific for low androstenone testis samples. Furthermore, we have selected SNPs in *IRG6, DSP, MX1* and *IFIT2* genes to validate their segregation and association in our population (Table S3 and Table S4). Polymorphisms in *IRG6* (g.118838598G>A), *MX1* (g.144420441C>T) and *IFIT2* (g.106102335 G>T) were associated with androstenone level (Table 9).

**Table 7:** Polymorphisms detected in testis samples.

Refseq ID	Gene name	SSC	Position	dbSNP	Reference	Alternate	Quality	RMSPS^a^	SNP Classification	Sample group
XM_003124689	HBA2	3	35253219	0	G	GA	333.66	39.10	Insertion	High and Low
XM_003124689	HBA2	3	35253521	0	G	GCTC	617.84	39.89	Insertion	High and Low
NM_213817	IRG6*	3	118838598	0	G	A	181.62	37.00	Synonymous	Low androstenone
XM_003124870	LOC100516362	3	48107044	0	G	GT	260.38	38.59	Insertion	High androstenone
XM_001928325	CD244	4	93149337	0	T	A	84.61	36.72	Synonymous	High androstenone
XM_003128168	DSP	7	4940734	0	G	A	31.18	37.00	Synonymous	Low androstenone
XM_003128168	DSP*	7	4944881	0	C	T	69.47	37.00	Synonymous	Low androstenone
XM_001928679	ARG2	7	99786827	0	A	AT	106.28	39.51	Insertion	High androstenone
NM_214061	MX1	13	144402807	0	A	G	43.93	37.40	Non- synonymous	High androstenone
NM_214061	MX1*	13	144420441	0	C	T	59.51	37.00	Non- synonymous	Low androstenone
XM_001928671	IFIT2*	14	106102335	rs80925743	G	T	98.81	37.00	Synonymous	Low androstenone
XM_001928671	IFIT2	14	106102694	0	G	A	40.63	37.00	Synonymous	Low androstenone

* the SNP validated in boar population using RFLP (see Table 9).

a Root mean square phred score.

**Table 8:** Polymorphisms detected in liver samples.

Refseq ID	Gene name	SSC	Position	dbSNP	Reference	Alternate	Quality	RMSPS^a^	SNP Classification	Sample group
XM_001928594	FMO5*	4	104473018	rs80837900	G	A	112.94	37.02	Synonymous	Low androstenone
NM_001005352	CYP7A1	4	77195279	0	G	A	1373.52	37.15	Non-synonymous	Low androstenone
NM_001005352	CYP7A1	4	77195397	0	T	C	4026.2	36.93	Synonymous	High and Low
NM_001005352	CYP7A1	4	77197364	0	T	C	1624.62	37.00	Synonymous	High and Low
NM_001005352	CYP7A1	4	77199510	0	A	T	416.26	37.00	Synonymous	Low androstenone
NM_001005352	CYP7A1	4	77199576	0	T	G	242.3	36.07	Synonymous	Low androstenone
NM_001005352	CYP7A1	4	77200294	0	G	A	274.6	37.00	Synonymous	Low androstenone
NM_001005352	CYP7A1	4	77200408	rs80923210	A	G	494.32	36.44	Synonymous	Low androstenone
NM_001005352	CYP7A1*	4	77201533	rs196960866	A	G	1880.98	37.00	Synonymous	Low androstenone
NM_001159615	KRT8	5	16715238	rs80814240	C	T	2365.07	36.65	Synonymous	High and Low
NM_001159615	KRT8	5	16718099	rs80845521	C	T	2186.76	37.01	Synonymous	High and Low
NM_001159615	KRT8	5	16720725	0	G	GT	1160.84	39.98	Insertion	High and Low
NM_001159615	KRT8	5	16721108	0	GGT	G	355.1	38.63	Deletion	High and Low
NM_001159615	KRT8	5	16721708	rs55618932	T	C	2941.94	36.61	Synonymous	High and Low
NM_001159615	KRT8	5	16721831	rs80916149	A	G	9343.88	37.02	Synonymous	High and Low
XM_003126180	KRT18*	5	16788495	0	G	A	327.13	37.16	Synonymous	Low androstenone
XM_003126180	KRT18	5	16789240	0	G	GA	304.54	38.77	Insertion	High and Low
XM_003126180	KRT18	5	16789379	rs81211893	G	A	596.36	36.52	Synonymous	High and Low
XM_003126180	KRT18	5	16789412	rs81211894	A	G	2116.22	36.56	Synonymous	High and Low
XM_003126180	KRT18	5	16789808	rs81211895	G	C	2188.63	36.89	Synonymous	High and Low
XM_003126180	KRT18	5	16789954	0	A	ATC	926.56	35.84	Insertion	High Androstenone
XM_001925061	HAL	5	82556747	0	G	GT	80.57	40.20	Insertion	High Androstenone
XM_001928022	HIST1H4K	7	22186329	0	C	T	717.72	33.45	Synonymous	Low androstenone
XM_001929558	CDKN1A	7	36992673	rs80964639	A	G	544.05	37.00	Synonymous	High Androstenone
XM_001929558	CDKN1A	7	36992792	0	A	G	214.41	37.00	Synonymous	High and Low
XM_003129674	TSKU	9	10759263	0	G	A	127.72	36.56	Synonymous	Low androstenone
NM_001123146	NNMT	9	40584781	0	G	GA	460.61	42.34	Insertion	High and Low
NM_214125	MBL2	14	101464163	0	C	T	236.74	38.58	Synonymous	High and Low
NM_214125	MBL2	14	101464174	0	T	G	624.35	38.02	Synonymous	High and Low
NM_214125	MBL2	14	101464216	0	A	T	2136.83	37.43	Synonymous	High and Low
NM_214125	MBL2	14	101464268	0	A	G	2123.64	37.42	Synonymous	High and Low
NM_214125	MBL2	14	101464309	0	T	C	1038.03	37.83	Synonymous	High and Low
NM_214125	MBL2	14	101464842	0	A	ACT	1693.34	37.6	Insertion	High and Low
NM_214125	MBL2	14	101467788	0	A	G	4598.53	36.97	Synonymous	High and Low
XM_001928302	SDS	14	38865735	0	G	GT	194.2	39.81	Synonymous	High Androstenone
XM_001928302	SDS	14	38868514	0	C	T	51.13	37.00	Non- synonymous	Low androstenone

* the SNP validated in boar population using RFLP (see Table 9).

a Root mean square phred score.

**Table 9:** Genotype and association analysis of selected candidate genes with androstenone.

Polymorphism	Boar taint compound [Ln(µg/g)]	Genotype (μ ± S.E.)			Effect (μ ± S.E.)	
		GG	GA	AA	Additive	Dominance
IRG6 G>A	Androstenone	4.93±0.30^c^	5.95±0.21^d^	5.82±0.10^d^	−0.44±0.15**	−0.57±0.23*
		CC	CT	TT		
DSP C>T	Androstenone	5.76±0.21	6.06±0.18	5.94±0.20	−0.09±0.08	−0.20±0.13
		CC	CT	TT		
MX1 C>T	Androstenone	5.73±0.12^a^	5.92±0.11^a^	5.30±0.18^b^	0.21±0.11	−0.40±0.14**
		GG	GT	TT		
IFIT2 G>T	Androstenone	6.27±0.18^a^	5.63±0.18^b^	5.61±0.10^b^	0.32±0.09**	0.31±0.19
		GG	GA	AA		
FMO5 G>A	Androstenone	5.96±0.27^a^	5.48±0.27^b^	5.97±0.20^a^	0.01±0.15	0.47±0.20*
		AA	AG	GG		
CYP7A1 A>G	Androstenone	4.63±0.32^a^	5.60±0.22^b^	5.73±0.08^b^	−0.55±0.16 **	−0.42±0.26
		GG	GA	AA		
KRT18 G>A	Androstenone	5.70±0.01*^c^*	5.82±0.23^c^	4.59±0.30^d^	−0.55±0.15 **	−0.66±0.25*

a,b <0.05; c,d<0.01; e,f <0.001;

* p<0.05;

** p<0.01.

Thirty six mutations were identified in 11 DEGs in liver samples (Table 8). Variation in *HAL* gene was specific for high androstenone liver samples whereas *FMO5*, *HIST1H4K* and *TSKU* gene variations were specific for low androstenone liver samples (Table 8). Read counts for individual samples for identified polymorphisms in testis and liver tissues are given in Table S2. Additionally, we have validated SNPs in highly polymorphic genes *CYP7A1, KRT18* and *FMO5* and their association in our population (Table S3 and Table S4). The SNP in *CYP7A1* (g.77201533 A>G), *KRT18* (g.16788495 G>A) and *FMO5* (g.104473018 G>A) were found to be associated with the phenotype androstenone level (Table 9).

## Discussion

### Analysis of RNA-Seq Data

The present study describes the transcriptome profiles of testis and liver for androstenone by using RNA-Seq. To the best of our knowledge this study provides the first comprehensive insight into the transcriptome of androstenone metabolism in testis and liver tissue by using RNA-Seq. Using the whole transcriptome sequencing technique, we were able to identify the levels of differentially expressed genes and associate these genes with divergent androstenone levels in terms of boar taint. Our findings clearly demonstrated the power of RNA-Seq and provide further insights into the transcriptome of testis and liver in androstenone at a finer resolution. Illumina sequencing data have been described as replicable with relatively little technical variation [35].

Although 45% to 50% (Table 1 and 2) of the fragments do not map to annotated exons in our study, we were able to identify genes associated with divergent androstenone levels. Porcine annotation is incomplete, as evidenced by read mapping annotation. The percentage of annotated reads varies from 15.6% to 60.8% in similar porcine transcriptome studies [36]; [37]; [38]. The differences between mapping percentages might be due to several factors such as primer biases, GC content, dinucleotide fragmentation sites, independent cell types and laboratory protocols [39]; [40]. Another factor is that the current reference transcriptome assembly might not cover all transcribed mRNA and consequently low abundant transcripts or rare alternative splicing isoforms are less likely to be mapped to transcriptome assembly [38].

### Differential Gene Expression and Gene Polymorphism Analysis in Testis

In this study, 46 genes were differentially regulated in testis with divergent androstenone levels (Table 3). Our findings of differential gene expression are in accordance with the current understanding of androstenone metabolism as well as the previous findings in functional studies. In our study, the most up and down regulated genes *DKK2* and *KRT82* were found to be novel genes related to androstenone metabolism. Dickkopf-related protein 2 is encoded by the *DKK2* gene which was identified as the highest up regulated gene in our study (Table 3). *DKK2* can act as either an agonist or antagonist of Wnt/beta-catenin signalling [41]. WNT signalling in the testis has not been well understood, however it has been shown to play an important role in proliferation and self-renewal of mouse and human spermatogonia [42]. Mutation in β-catenin leads to the over activity of β-catenin in Sertoli cells caused testicular cord disruption, germ cell depletion, and inhibition of Müllerian duct regression suggesting that inhibition of β-catenin signalling is essential for Sertoli cell and testicular cord maintenance and germ cell survival [43]. Baes et al. [44] found that breeding against androstenone may have slightly adverse effects on semen quality. In the light of these external references it could be speculated that up regulation of *DKK2* gene in this study may have antagonistic effect on Wnt/beta-catenin signalling pathway which has shown to cause negative effects on sperm production. *KRT82* was the highest down regulated gene in high androstenone testis tissues in our study (Table 3). The protein encoded by this gene is a member of the keratin gene family which contains at least 54 functional keratin genes in humans [45]. Keratin-related genes are known to be affected by androgen exposure, especially by Dihydrotestosterone (DHT) exposure [46]. Relation of DHT with androsterone has been shown by Rizner et al. [47]. These literature evidences show that down-regulation of *KRT82* gene is the end result of high androgen metabolism in testis and not directly involved in androsterone synthesis.

There are similarities between gene expression differences found with RNA-Seq and those reported in previous microarray studies in porcine testis and liver tissues [27]; [28]; [29]. Grindflek et al. [48] and Moe et al. [29] reported cytochrome P450 superfamily genes to be differentially regulated in their investigated testis samples. In our study, other members of cytochrome P450 family genes were found to be differentially regulated in addition to genes reported by these previous studies. Our findings showed that genes *CYP4B1*, *CYP4A11* and *CYP2C33* were up regulated and gene *CYP2B22* was down regulated (Table 3). Among these genes, *CYP4A11* was enriched in Gene Ontology categories molecular transport, small molecule biochemistry and lipid metabolism and gene *CYP4B1* was revealed in small molecule biochemistry (Table 5). In accordance with our results, Moe et al. [29] also showed lipid metabolism to be one of the enriched GO categories for DEGs in testis samples.

In addition to transcriptome quantification, RNA-Seq technology provides valuable information regarding gene polymorphisms which could be directly correlated with the relevant phenotype. Several holistic gene expression analyses have been performed for boar taint compounds by using microarray or Real-Time PCR technology [27]; [28]; [29]. Our study extends these observations by correlating differentially regulated genes with associated polymorphisms. Gene polymorphisms in the exonic regions might have direct effect on the expression of transcripts and connecting our identified polymorphisms from RNA deep sequencing with GWAS studies may give additional insight to variation in the androstenone levels. Results in our study revealed 12 mutations in androstenone testis samples (Table 7). On SSC3 four polymorphism were identified, two at 35 Mb (insertion) on gene *HBA2*, one at 48 Mb (SNP) on gene *LOC100516362* and one at 118 Mb (SNP) on gene *IRG6* (Table 7). Grindflek et al. [2] found two QTL regions on the same chromosome for androstenone at 38 to 40 Mb in Duroc breed by using SNP chip genotyping which is in agreement with our results. Similar to our detected SNP at 118 Mb, an androstenone QTL on SSC3 was identified between 113 Mb and 122 Mb regions in Duroc, Landrace and Yorkshire breeds [23]. We identified a SNP on SSC4 at position 93 Mb on gene *CD244*, however, no previous QTL region associated with boar taint related traits was reported before. Three polymorphisms were identified on SSC7, two SNPs on gene *DSP* at 4.9 Mb and an insertion polymorphism at 99 Mb on gene *ARG2*. SNP genotyping study by Grindflek et al. identified an androstenone related QTL region on chromosome 7 between region 80.8 Mb and 88.3 Mb [2] which is in close proximity to the polymorphism detected on gene *ARG2* in this study. Additionally, Ren et al. [49] identified a male reproductive trait (testosterone level) related QTL on the same chromosome at 77.2 cM region. In our study, two SNPs were identified on gene *MX1* at position 144 Mb on SSC13, yet to the best of our knowledge no QTL regions related with boar taint or male reproductive traits has been reported in this region. On SSC14 at position 106 Mb, two SNPs were identified on gene *IFIT2*. Cross matching the chromosomal positions of these SNPs with data from dbSNP database showed that one of the SNPs (at position 106,102,335) has already been annotated in the SNP database (dbSNP ID: rs80925743). A QTL region for androstenone was identified on the same chromosome between 87.9 cM and 108.7 cM by Lee et al. [24] and the SNPs identified in our study fit into this previously identified androstenone QTL region.

### Differential Gene Expression and Gene Polymorphism Analysis in Liver

Twenty five genes were found to be differentially regulated in liver tissue with divergent androstenone levels (Table 4). The top two up regulated genes in our liver sample were *LOC100512122* with log fold change 3.89 and *LOC100511195* with log fold change 3.57. However, we were not able to identify either the gene names or function through orthologue databases or BLAST sequence similarity searches. As a result, the functions of these genes cannot be discussed in detail here.

*IP6K1* was the third highest up regulated in our liver samples. Inositol hexakisphosphate kinase 1 (*IP6K1*) is a member of the inositol phosphokinase family which encodes protein responsible for the conversion of inositol hexakisphosphate (InsP6) to diphosphoinositol pentakisphosphate (InsP7/PP-InsP5) [50]. Chakraborty et al. [51] have shown that targeted deletion of *IP6K1* in mice liver has increased Akt and mTOR signalling and decreased GSK3β signalling. Since this gene is highly expressed in liver, several factors including the diet of the sample population might have a larger impact on the expression of this gene. At this point, we are not able to pinpoint the effect of this gene (*IP6K1*) on androstenone metabolism in liver.

In our liver sample, *HSD17B2* was the highest down regulated gene with fold change −2.86 (Table 4). Hydroxysteroid (17-beta) dehydrogenase 2 (*HSD17B2*) regulate the availability of testosterone and androstenedione in tissues by catalysing interconvertion of active and inactive forms of steroids [52]. Gene expression studies by Moe et al. [28] have also shown the down regulation of *HSD17B2* gene in liver sample. Moreover, different members of the HSD enzyme family (*HSD17B4*, *HSD17B11* and *HSD17B13*) were found to be differentially regulated in Duroc and Norwegian Landrace populations [28].

Our results showed that cytochrome P450 family gene *CYP7A1* is differentially regulated in liver samples. *CYP7A1* is the rate-limiting enzyme in the synthesis of bile acid from cholesterol. The conversion of cholesterol to bile acid is the major pathway for cholesterol metabolism [30]. *CYP7A1* is a cytochrome P450 heme enzyme that oxidizes cholesterol using molecular oxygen. Down-regulation of *CYP7A1* causes reduced fat catabolism in liver which may lead to higher fat accumulation and androstenone level due to the dynamic relationship between androstenone in plasma and adipose tissue [53]. Gene expression profiles by Moe et al. [28] have also shown cytochrome P450 family genes to be differentially regulated in liver samples.

Another gene family found to be differentially expressed in our transcriptome analysis is flavin-containing monooxygenases (FMOs) gene family. The FMO family of enzymes converts lipophilic compounds into more polar metabolites and decreases activity of the compounds [54]. In the study conducted by Moe et al. [28], using microarray analysis, *FMO1* is reported to be up-regulated in higher androstenone pigs. In contrast, *FMO5* was found to be down-regulated in high androstenone liver samples in our study. Since androstenone is a lipophilic compound, we speculate that androstenone level was negatively correlated with *FMO5* activity. Since androstenone is a lipophilic compound, we speculate that androstenone level was negatively correlated with *FMO5* activity.

Among the differentially expressed genes in liver, the gene *CDKN1A* was enriched in GO categories amino acid metabolism, small molecule biochemistry, lipid metabolism and molecular transport. The differentially expressed gene *CYP7A1* was enriched in GO categories such as small molecule biochemistry, lipid metabolism and molecular transport. Gene Ontology functional analysis by Moe et al [28] has also shown that the GO categories lipid metabolism and amino acid metabolism were enriched.

Gene polymorphism analysis has shown that there were thirty six mutations in 11 DEGs in liver samples (Table 8). Eight SNPs were identified on SSC4 at position 77 Mb (Table 8) which were mapped to gene *CYP7A1.* In close adjacency to this region Quintanilla et al. [25] identified an androstenone related QTL at position 72 cM. An additional SNP was identified on SSC4 at position 104 Mb mapped to gene *FMO5* however, this position was not mapped as androstenone related QTL region by any previous studies. Our results have also shown that this SNP at position 104,473,018 has been already reported in dbSNP database (dbSNP ID: rs80837900) (Table 8). Six polymorphisms on SSC5 at position 16.7 Mb were mapped to gene *KRT8*. Out of these identified polymorphisms, four SNPs were previously mapped to dbSNP database (Table 8). Another set of 6 polymorphisms on SSC 5 at position 16.7 Mb mapped to the gene *KRT18*. Three SNPs among these six polymorphisms were already identified and reported in dbSNP database (Table 8). Grindflek et al. [2] detailed an androstenone QTL region on SSC5 between 20.4 and 22.2 Mb in close proximity to our reported polymorphisms (Table 8). An insertion gene polymorphism at position 82 Mb on the same chromosome mapped to *HAL* gene was also indentified in our study. On SSC 7 we identified 3 SNPs, one at position 22 Mb on gene *HIST1H4K* and two at position 36 Mb mapped to gene *CDKN1A*. One of the SNP mapped to *CDKN1A* at position 36.9 Mb has already been reported in dbSNP database (dbSNP ID: rs80964639). An androstenone QTL region on SSC7 between position 33.6 and 88.3 Mb was already described by Grindflek et al. [2]. Two SNPs on *CDKN1A* identified in our study falls into this previously mentioned QTL region. Genome wide association study by Grindflek et al. [2] described androstenone related QTL region on SSC9 at position 7.5 to 8.0 Mb. We report an SNP on the same chromosome at position 10 Mb, close to the previously reported QTL region (Table 8). In addition, we obtained an insertion polymorphism mapped to *NNMT* gene at position 40 Mb. We identified two polymorphisms at 38 Mb on *SDS* gene in the vicinity of the androstenone QTL region on SSC14 at 37 cM [25]. Furthermore, our analysis revealed 7 additional SNPs on SSC14 at 101 Mb on *MBL2* gene. Lee et al. [24] described a QTL on SSC14 at position 87.9 to 108.7 cM for androstenone in the Large White × Meishan crossbred population.

Selected polymorphisms in genes *IRG6, MX1, IFIT2, FMO5, CYP7A1* and *KRT18* were found to be associated with the phenotype androstenone level in this study (Table 9). An association study was performed for a SNP (g.494 A>G) in the *FMO5* gene but no statistical relation could be detected with the off flavour score in the Berkshire x Yorkshire resource population [55]. Location of *IFIT2* gene on SSC14 incorporated the QTL affecting androstenone in Yorkshire pig [56] and subjective pork flavour in Large White and Meishan pigs [24]. *MX1* is an interesting candidate gene for disease resistance in farm animals [57] but this study first identifies association with boar taint compounds. No study investigated association of *CYP7A1* with boar taint compounds. Some study reported association of this gene with plasma cholesterol in pigs [58]. Boar taint is related to the adipose tissues since lean pigs have low boar taint compounds [59]. The function of highly polymorphic *KRT18* is relating to pathological processes in liver but involvement in boar taint is not quite clear. However, this gene maps close to a region on SSC5 affecting androstenone in pigs [2].

### Conclusion

Here we showed whole genome expression differences for varying androstenone levels in testis and liver tissues. RNA-Seq provided high resolution map of transcriptional activities and genetic polymorphisms in these tissues. However, due to incomplete porcine annotations, only around 50% of the total reads could be mapped to annotated references. The improvements in pig genome annotations may lead to better coverage and detailed understanding of genetic and functional variants such as novel transcripts, isoforms, sequence polymorphisms and non-coding RNAs. Integration of high throughput genomic and genetic data (eQTL) with proteomic and metabolomic data can provide additional new insight into common biological processes and interaction networks responsible for boar taint related traits.

On the basis of number of DEGs, our results confirm that transcriptome activity in testis is higher in comparison to liver tissue for androstenone biosynthesis. These results also show that the entire functional pathway involved in androstenone metabolism is not completely understood and through this study, we propose additional functional candidate genes such as, *DKK2* and *CYP2B22* in testis and *IP6K1* and *HSD17B2* in liver for androstenone metabolism. Importantly, most of the DEGs are in QTL positions functionally related to pathways involved in boar taint. Furthermore, various gene polymorphisms were also detected in testis and liver DEGs and associations are validated with androstenone levels. Potential polymorphisms and association were identified in DEGs such as *IRG6, MX1,* and *IFIT2* in testis and *CYP7A1*, *FMO5* and *KRT18* in liver. This transcriptome and polymorphisms analysis using RNA deep sequencing combining with association analysis has revealed potential candidate genes affecting boar taint compound. It is speculated that these polymorphisms could be used as biomarkers for boar taint related traits. However, further validation is required to confirm the effect of these biomarkers in other animal populations.

## Materials and Methods

### Animals and Phenotypes

Tissue samples and phenotypes were collected from the Duroc × F_2_ cross animals. F_2_ was created by crossing F_1_ animals (Leicoma × German Landrace) with Large White pig breed. Duroc × F_2_ boars were on average 116 days old and had on average 90 kg live weight when slaughtered. All pigs were slaughtered in commercial abattoir, called Landesanstalt für Schweinezucht – LSZ Boxberg. Slaughterhouse management gave the necessary permissions for the tissue and organ collections. Animals were bred and growth, carcass and meat quality data were collected according to guidelines of the German performance test [60]. Tissue samples from testis and liver were frozen in liquid nitrogen immediately after slaughter and stored at −80°C until used for RNA extraction. Fat samples were collected from the neck and stored at −20°C until used for androstenone measurements. For the quantification of androstenone an in-house gas-chromatography/mass spectrometry (GC-MS) method was applied as described previously [61]. Pigs having a fat androstenone level less than 0.5 µg/g and greater than 1.0 µg/g were defined as low and high androstenone samples, respectively. Ten boars were selected from a pool of 100 pigs and the average androstenone value for these selected animals was 1.36±0.45 µg/g. Notably, these 100 boars were used for association study (Table S3 and Table S4). RNA was isolated from testis and liver of 5 pigs with extreme high (2.48±0.56 µg/g) and 5 pigs with extreme low levels of androstenone (0.24±0.06 µg/g). Total RNA was extracted using RNeasy Mini Kit according to manufacturer’s recommendations (Qiagen). Total RNA was treated using on-column RNase-Free DNase set (Promega) and quantified using spectrophotometer (NanoDrop, ND8000, Thermo Scientific). RNA quality was assessed using an Agilent 2100 Bioanalyser and RNA Nano 6000 Labchip kit (Agilent Technologies).

### Library Construction and Sequencing

Full-length cDNA was obtained from 1 µg of RNA, with the SMART cDNA Library Construction Kit (Clontech, USA), according to the manufacturer’s instructions. Libraries of amplified RNA for each sample were prepared following the Illumina mRNA-Seq protocol. The library preparations were sequenced on an Illumina HiSeq 2000 as single-reads to 100 bp using 1 lane per sample on the same flow-cell (first sequencing run) at GATC Biotech AG (Konstanz, Germany). All sequences were analysed using the CASAVA v1.7 (Illumina, USA). The deep sequencing data have been deposited in NCBI SRA database and are accessible through GEO series accession number GSE44171 (http://www.ncbi.nlm.nih.gov/geo/query/acc.cgi?acc=GSE44171).

### Reference Sequences and Alignment

Two different reference sequence sets were generated from NCBI Sscrofa 9.2 assembly: (1) the reference sequence set generated for differential expression analysis comprised of RefSeq mRNA sequences (cDNA sequences) and candidate transcripts from NCBI UniGene database (Sscrofa). (2) For gene variation analysis a different reference sequence set, generated from whole genome sequence (chromosome assembly) was used. During sequencing experiment, Sscrofa NCBI 10.2 assembly was not released and Sscrofa 9.2 covered ∼8.5 K unannotated SNPs (dbSNP database). The released Sscrofa 10.2 assembly consists of 566 K SNP http://www.ncbi.nlm.nih.gov/Taxonomy/Browser/wwwtax.cgi?mode=Info&id=9823 (Accession date 4/02/2013) with annotation information for 460 K SNP (dbSNP database). In order to make use of this enriched SNP information, we used NCBI Remap tool (http://www.ncbi.nlm.nih.gov/genome/tools/remap) to convert Sscrofa 10.2 SNP genomic positions to Sscrofa9.2 positions. Using the whole chromosomal assembly for read mapping in gene variation analysis step allowed us to match the polymorphisms identified in this analysis with polymorphisms available for porcine genome in dbSNP database based on chromosomal positions. Raw reads were mapped to reference sets using BWA algorithm (http://bio-bwa.sourceforge.net/) with the default parameters [62].

### Differential Gene Expression Analysis

The differential gene expression analysis was designed to contrast the difference in the expression of genes between high and low androstenone samples. For differential gene expression analysis with raw count data a R package DESeq was used [33]. The normalization procedure in DESeq handles the differences in the number of reads in each sample. For this purpose, DESeq first generates a fictitious reference sample, with read counts defined as the geometric mean of all the samples. The read counts for each gene in each sample is divided by this geometric mean to obtain the normalized counts. To model the null distribution of the count data, DESeq follows an error model that uses the negative binomial distribution, with variance and mean linked by local regression. The method controls type-I error and provides good detection power [33]. After analysis using DESeq, DEGs were filtered based on *p*-adjusted value [63] 0.05 and fold change ≥1.5. Additionally, the gene expression data was also analyzed using a Generalized Linear Model (GLM) function implemented in DESeq to calculate both within and between group deviances. As sanity checking and filtration step, we cross matched the results from both analysis (*p*-adjusted ≤0.05 and fold change ≥1.5 criteria and GLM analysis) and only those genes which appeared to be significant in both the tests (*p*-value ≤0.05), were selected for further analysis. The results of GLM analysis are given in Table S2.

### Gene Variation Analysis

For gene variation analysis the mapping files generated by aligning the raw reads to reference sequence set (2) were used. All the downstream analysis was performed using Genome Analysis Toolkit (GATK) [64] and Picard Tools (http://picard.sourceforge.net/). The Genome Analysis Toolkit (GATK) was used for local realignment incorporating Sscrofa 9.2 converted SNPs which was described in the previous section. SNPs were furthermore classified as synonymous or non-synonymous using the GeneWise software (http://www.ebi.ac.uk/Tools/psa/genewise/last accessed 21.03.2013) by comparing between protein sequence and nucleotides incorporated SNP position [65]. Covariate counting and base quality score recalibration were done using the default parameters suggested by GATK toolkit. The re-aligned and recalibrated mapping files were grouped according to tissue and phenotype categories. Variant calling was performed for each group using GATK UnifiedGenotyper [64]. To find out the differentially expressed genes that also harboured sequence polymorphisms, we filtered the results from UnifiedGenotyper with chromosomal positions of DEGs and retained only those which mapped to DEG chromosomal positions. By this way, we were able to isolate a handful of mutations that mapped to DEGs from many thousands of identified potential sequence polymorphisms. Additionally to understand whether these identified polymorphisms segregate either in only one sample group (high androstenone or low androstenone group) or in both the groups (high and low androstenone group) we calculated the read/coverage depth of these polymorphisms in all the samples. The results of this analysis are detailed in the results section and read coverage for individual samples are given in Table S2.

### Pathways and Networks Analysis

A list of the DEGs was uploaded into the Ingenuity Pathway Analysis (IPA) software (Ingenuity Systems, www.ingenuity.com) to identify relationships between the genes of interest and to uncover common processes and pathways. The ‘Functional Analysis’ tool of the IPA software was used to identify the biological functions that were most significant to the data set [5].

### Quantitative Real-Time PCR (qRT-PCR) Analysis

For qRT-PCR experiment, total RNA from testis and liver samples were isolated from the 10 boars used for deep sequencing. Additionally, RNA was isolated from the similar tissues of 10 independent boars with divergent androstenone level among the remaining 90 boars. cDNA were synthesised by reverse transcription PCR using 2 µg of total RNA, SuperScript II reverse transcriptase (Invitrogen) and oligo(dT)12 primer (Invitrogen). Gene specific primers for the qRT-PCR were designed by using the Primer3 software [66]. Detailed information for primers used in this study was given in Table 10. In each run, the 96-well microtiter plate contained each cDNA sample and no-template control. The qRT-PCR was conducted with the following program: 95°C for 3 min and 40 cycles 95°C for 15 s/60°C for 45 s on the StepOne Plus qPCR system (Applied Biosystem). For each PCR reaction 10 µl iTaqTM SYBR® Green Supermix with Rox PCR core reagents (Bio-Rad), 2 µl of cDNA (50 ng/µl) and an optimized amount of primers were mixed with ddH_2_O to a final reaction volume of 20 µl per well. All samples were analysed twice (technical replication) and the geometric mean of the Ct values were further used for mRNA expression profiling. The geometric mean of two housekeeping genes *GAPDH* and *PPIA* were used for normalization of the target genes. The delta Ct (ΔCt) values were calculated as the difference between target gene and geometric mean of the reference genes: (ΔCt = Ct_target_− Ct_housekeeping genes_) as described in Silver et al. [67]. Final results were reported as fold change calculated from delta Ct-values. Details of primers which were used for qRT-PCR study are shown in Table 10.

**Table 10:** Details of primers used for qRT-PCR analysis and genotyping.

Gene	Reference ID	Primer sequences (5′→3′)	Application	Position*	Enzyme	Annealing temperature (°C)	Product size (bp)	RFLP-patterns
CYP2B22	NM_214413.1	F: CACCACCATCCTCCAGAACT R: GGCAGGAACTGGATCTGGTA	qRT-PCR	–	–	52	120	–
ARG2	XM_001928679.2	F: GGAAGCTGGCTTGATGAAAA R: CCACTGAGCGAGGATTCACT	qRT-PCR	–	–	55	128	–
MSMO1	NM_213752.1	F: CCTGGCACTATTTCCTGCAT R: TAGGGTTTCCAGAGGGTGTG	qRT-PCR	–	–	55	128	–
EDN1	NM_213882.1	F: TTCAGGGAGAAACACCCAAG R:CGAGACGGAAGAAAGCAAAG	qRT-PCR	–	–	55	121	–
CYP2C33	NM_214414.1	F: AGCTGTGCCTCATCCCTAGA R: GTGTTTCTGTCCCAGGCAAT	qRT-PCR	–	–	56	133	–
IP6K1	XM_001925759.2	F: CTGCCAGCCTGTGTCTGTAA R: ATGGCACCAGAATCAGAAGG	qRT-PCR	–	–	55	136	–
BTG3	XM_003132741.1	F: CCAGGAATGTACCGAGGAAA R: ACAATGCATTCCAGGAGGAG	qRT-PCR	–	–	55	138	–
CYP7A1	NM_001005352.2	F: TTCCCGATTCATGTGTTCAA R: ACCAGTTCCGAGATGTGGTC	qRT-PCR	–	–	54	104	–
FMO5	XM_001928594.1	F:GGCCTGAAGCCTAAACACAG R:CCTGGAGCCATCCTCAAATA	qRT-PCR	–	–	55	147	–
HSD17B2	NM_001167649.1	F: TGCAGAACAGAGGACTGTGG R: GCCATGCATCGTTTGTATTG	qRT-PCR	–	–	54	103	–
PPIA	NM_214353	F: CACAAACGGTTCCCAGTTT R: TGTCCACAGTCAGCAATGGT	qRT-PCR	–	–	58	171	–
GAPDH	AF017079	F:ACCCAGAAGACTGTGGATGG R:ACGCCTGCTTCACCACCTTC	qRT-PCR	–	–	60	247	–
IRG6	NM_213817	F: CTGGTACCTGTCACCTTTGC R: GGGTGAAGTGGTAATTGACG	Genotyping	Exon 3	HaeIII	60	232	GG: 154+78AA: 232
DSP	XM_003128168	F: AACCTGATTGATCGGGAAAC R: GCTGACCTTCTTTTTGGTGA	Genotyping	Exon 1	HpyCH4IV	55	207	CC: 109+98TT: 207
MX1	NM_214061	F:CACTTCCAAATGGAGCAGAT R:GACTCGCAGACTCACCTGAT	Genotyping	Exon 2	AciI	55	204	CC: 125+79TT: 204
IFIT2	XM_001928671	F:AAGAAGTTTTCCAGCCCCTA R:TTATCCAGACGGTAGCTTGC	Genotyping	Exon 3	DrdI	55	188	GG: 145+107TT: 252
FMO5	XM_001928594	F:AAAGGTTCGACCATGAAATG R: TATGGCAGCTGTCTCTGTGA	Genotyping	Exon 3	HpyCH4III	55	223	GG: 134+89AA: 223
CYP7A1	NM_001005352	F:TGTCCAGGAAATCAAGCAAT R:CGTCATCAGCTGTCCTCTTT	Genotyping	Exon 2	HpyCH4V	55	199	AA: 101+98GG: 199
KRT18	NM_001159615	F: GGGTTGAGAAGGTTCTGGAT R: CTCCTCGTGGTTCTTCTTCA	Genotyping	Exon 2	HpyCH4V	55	215	GG: 149+66AA: 215

* Position according to coding region in Sus scrofa.

### Validation of SNP and Association Study

Seven SNPs were selected covering both the testis and liver samples for further validation and association study (Table 9). Genotyping in 100 boars were performed by PCR-RFLP method. In brief, a working solution with a final concentration of 50 ng/µl DNA was prepared and stored at 4°C for further analysis. Polymerase chain reactions (PCR) were performed in a 20 µl volume containing 2 µl of genomic DNA, 1 × PCR buffer (with 1.5 mM MgCl_2_), 0.25 mM of dNTP, 5 pM of each primer and 0.1 U of Taq DNA polymerase (GeneCraft). The PCR product was checked on 1.5% agarose gel (Fischer Scientific Ltd) and digested by using the restriction enzyme (Table 10). Digested PCR-RFLP products were resolved in 3% agarose gels. Details of GenBank accession numbers, primers sequences, annealing temperature and SNP position used in this study are listed in Table 10. Statistical analyses were performed using SAS 9.2 (SAS Institute Inc., Cary, USA). Effects of slaughter age, husbandry system (pen) as well as genotype on boar taint compound androstenone were assessed with fixed effect model (ANOVA) using PROC GLM. For all models, fixed effects included genotype and pen (group, individual), and age of slaughter was fitted as a covariate for boar taint compound androstenone. Due to the skewed nature of the androstenone, data were transformed with natural logarithm before ANOVA to achieve normality. Least square mean values for the loci genotypes were compared by t-test and p-values were adjusted by the Tukey–Kramer correction [68]; [69]; [70].

## Supporting Information

Figure S1 **The smear plots for differential expression between high and low androstenone levels in testis and liver.**
(TIF)

Table S1 **GLM analysis results for testis and liver DEGs.**
(DOC)

Table S2 **Sample read counts for polymorphisms on testis and liver DEGs.**
(DOC)

Table S3 **Selected SNP detected by RNA-seq that were validated using RFLP.**
(DOC)

Table S4 **Genotype, allele frequencies and the chi-square test of selected SNPs validated using RFLP.**
(DOC)
