# Supplementary figures and images for: RNA Deep Sequencing Reveals Novel Candidate Genes and Polymorphisms in Boar Testis and Liver Tissues with Divergent Androstenone Levels
Source: PLoS One. 2013 May 16;8(5):e63259. doi: 10.1371/journal.pone.0063259 (PMC3655983; doi:10.1371/journal.pone.0063259)

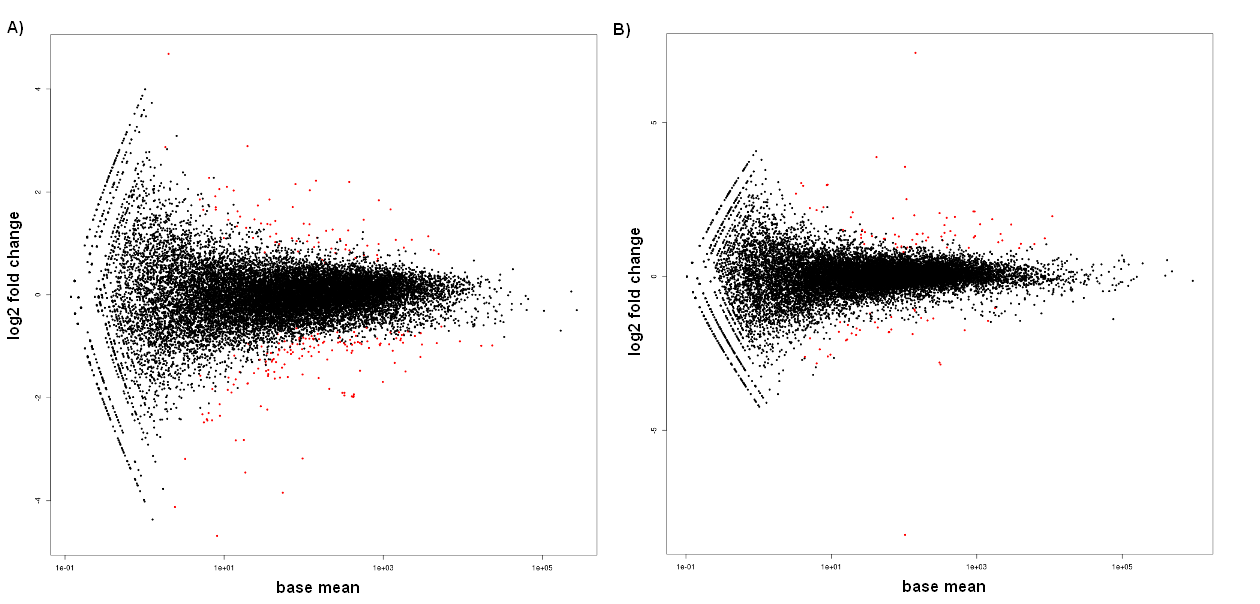

Supplement: Figure S1 — The smear plots for differential expression between high and low androstenone levels in testis and liver. (TIF) [file pone.0063259.s001.tif]
