# Supplementary material for: RNA Deep Sequencing Reveals Novel Candidate Genes and Polymorphisms in Boar Testis and Liver Tissues with Divergent Androstenone Levels
Source: PLoS One. 2013 May 16;8(5):e63259. doi: 10.1371/journal.pone.0063259 (PMC3655983; doi:10.1371/journal.pone.0063259)
Supplement: Table S1 — GLM analysis results for testis and liver DEGs. (DOC) [file pone.0063259.s002.doc]

**Table S1. GLM analysis results for testis DEGs**

| Gene | Reference ID | Total deviance | Within group deviance | Between group deviance | pvals.GLM | padj.GLM |
| --- | --- | --- | --- | --- | --- | --- |
| DKK2 | XM_003129269.1 | 94,57405344 | 49,01098891 | 45,56306453 | 1.48e-11 | 1.82e-08 |
| AMN | XM_001925648.2 | 56,13687537 | 41,18519362 | 14,95168175 | 0,00011 | 0,0224 |
| LOC100519550 | XM_003127761.1 | 219,1547338 | 157,6625993 | 61,49213449 | 4.44e-15 | 2.19e-11 |
| CYP4B1 | XM_003128017.1 | 187,8129657 | 121,4060867 | 66,40687909 | 3.33e-16 | 2.19e-12 |
| CD244 | XM_001928325.2 | 99,5360863 | 48,82215609 | 50,71393021 | 1.07e-12 | 1.54e-09 |
| ADAMTS4 | XM_001927507.2 | 59,06631207 | 41,80910286 | 17,25720921 | 3.26e-05 | 0,00882 |
| CYP4A11 | XM_003128032.1 | 149,5756533 | 100,5911754 | 48,98447792 | 2.58e-12 | 3.39e-09 |
| HAL | XM_001925061.1 | 49,77083672 | 31,15547537 | 18,61536134 | 1.6e-05 | 0,00538 |
| CYP2C33 | NM_214414.1 | 24,18405324 | 11,4387478 | 12,74530544 | 0,000357 | 0,0453 |
| AMY2B | XM_003125887.1 | 41,72791373 | 12,91784401 | 28,81006972 | 7.98e-08 | 4.92e-05 |
| ARG2 | XM_001928679.2 | 107,5216802 | 56,85667722 | 50,66500299 | 1.1e-12 | 1.54e-09 |
| LOC100516362 | XM_003124870.1 | 35,68008104 | 13,78452286 | 21,89555818 | 2.88e-06 | 0,00124 |
| LOC100521272 | XM_003126855.1 | 166,7184634 | 131,6674609 | 35,05100243 | 3.21e-09 | 2.53e-06 |
| MSMO1 | NM_213752.1 | 71,2762284 | 29,07879541 | 42,19743299 | 8.25e-11 | 8.14e-08 |
| KRT4 | XM_001927218.2 | 92,01257247 | 75,96178014 | 16,05079233 | 6.17e-05 | 0,015 |
| MPP7 | XM_003130762.1 | 43,08332655 | 20,92364435 | 22,15968221 | 2.51e-06 | 0,00115 |
| DSP | XM_003128168.1 | 103,5494219 | 74,14399114 | 29,40543073 | 5.87e-08 | 3.73e-05 |
| AMHR2 | XM_003126187.1 | 57,92658111 | 37,42076386 | 20,50581725 | 5.95e-06 | 0,00234 |
| SLA-3 | AB105388.1 | 142,0949217 | 111,8059984 | 30,28892327 | 3.72e-08 | 2.45e-05 |
| HAAO | XM_003125193.1 | 51,35356658 | 26,37997478 | 24,97359181 | 5.81e-07 | 0,000327 |
| MX1 | NM_214061.1 | 107,1777724 | 71,41987495 | 35,75789748 | 2.23e-09 | 1.84e-06 |
| MX2 | NM_001097416.1 | 103,425223 | 76,19198866 | 27,2332343 | 1.8e-07 | 0,000105 |
| IFIT2 | XM_001928671.2 | 46,35404599 | 31,18693736 | 15,16710863 | 9.84e-05 | 0,0209 |
| HBB | NM_001144841.1 | 101,7538105 | 58,47173398 | 43,28207657 | 4.74e-11 | 4.92e-08 |
| ARL4C | XM_003133753.1 | 23,53224016 | 6,019485451 | 17,51275471 | 2.85e-05 | 0,00853 |
| EDN1 | NM_213882.1 | 24,61048373 | 4,989608498 | 19,62087523 | 9.44e-06 | 0,00365 |
| HBM | XM_003124683.1 | 33,48467605 | 18,62044326 | 14,86423279 | 0,000116 | 0,0228 |
| HBD | XM_003129515.1 | 102,6293463 | 57,88556504 | 44,74378126 | 2.25e-11 | 2.61e-08 |
| HBA2 | XM_003124688.1 | 104,7535393 | 53,57587865 | 51,17766061 | 8.44e-13 | 1.51e-09 |
| HBA2 | XM_003124690.1 | 97,49026851 | 46,61657353 | 50,87369498 | 9.85e-13 | 1.54e-09 |
| HBA2 | XM_003124687.1 | 106,5499909 | 52,87307029 | 53,67692061 | 2.36e-13 | 4.68e-10 |
| HBA2 | XM_003124689.1 | 104,3856804 | 50,71524294 | 53,67043745 | 2.37e-13 | 4.68e-10 |
| HBA2 | XM_003124685.1 | 104,3751932 | 49,03679511 | 55,33839812 | 1.01e-13 | 2.86e-10 |
| HBA2 | XM_003124684.1 | 110,7205225 | 55,3359343 | 55,38458821 | 9.91e-14 | 2.86e-10 |
| HBA2 | XM_003124686.1 | 104,4061617 | 48,13099392 | 56,27516781 | 6.31e-14 | 2.49e-10 |
| FRK | XM_001925792.2 | 33,43199904 | 15,45709943 | 17,97489961 | 2.24e-05 | 0,0069 |
| IRG6 | NM_213817.1 | 128,465334 | 92,61463483 | 35,85069918 | 2.13e-09 | 1.83e-06 |
| SYT10 | XM_001927016.2 | 68,17520468 | 30,58017177 | 37,59503291 | 8.71e-10 | 7.8e-07 |
| S100A2 | XM_001929559.1 | 47,37447874 | 26,54715015 | 20,8273286 | 5.03e-06 | 0,00206 |
| CD5 | XM_003122679.1 | 22,08153823 | 7,920153303 | 14,16138493 | 0,000168 | 0,0282 |
| CYP2B22 | NM_214413.1 | 25,33892965 | 11,22154026 | 14,11738939 | 0,000172 | 0,0282 |
| CYTL1 | XM_003128849.1 | 120,4327297 | 87,45572125 | 32,97700847 | 9.33e-09 | 6.81e-06 |
| S100A2 | XM_001929556.1 | 78,45311566 | 39,61130608 | 38,84180959 | 4.6e-10 | 4.32e-07 |
| CHRNA3 | XM_001925760.2 | 122,9108731 | 68,39829968 | 54,51257342 | 1.54e-13 | 3.81e-10 |
| OLFRA03 | XM_001926523.1 | 23,41749837 | 9,255928537 | 14,16156983 | 0,000168 | 0,0282 |
| KRT82 | XM_003126157.1 | 84,08300443 | 39,50775683 | 44,57524759 | 2.45e-11 | 2.68e-08 |

**Table S1. GLM analysis results for liver DEGs**

| Gene | Reference ID | Total deviance | Within group deviance | Between group deviance | pvals.GLM | padj.GLM |
| --- | --- | --- | --- | --- | --- | --- |
| LOC100512122 | XM_003130359.1 | 156,05915 | 96,172635 | 59,8865151 | 1.01e-14 | 4.39e-11 |
| LOC100511195 | XR_115925.1 | 131,766902 | 71,0144286 | 60,7524731 | 6.44e-15 | 3.73e-11 |
| IP6K1 | XM_001925759.2 | 38,8051314 | 23,3454923 | 15,4596391 | 8.43e-05 | 0,0473 |
| AMPD3 | XM_003135226.1 | 47,2594532 | 25,7020468 | 21,5574064 | 3.43e-06 | 0,00391 |
| LOC100521668 | XR_116002.1 | 90,437071 | 56,4002305 | 34,0368405 | 5.41e-09 | 1.34e-05 |
| SDS | XM_001928302.2 | 53,1108229 | 26,0579749 | 27,0528481 | 1.98e-07 | 0,000382 |
| BTG3 | XM_003132741.1 | 60,5632263 | 33,5013371 | 27,0618892 | 1.97e-07 | 0,000382 |
| KRT78 | XM_001927194.2 | 97,8180328 | 78,8485531 | 18,9694797 | 1.33e-05 | 0,0122 |
| SMPDL3A | XM_003121227.1 | 64,3571135 | 41,7138815 | 22,6432319 | 1.95e-06 | 0,00261 |
| KRT8 | NM_001159615.1 | 76,3422238 | 52,6410569 | 23,7011669 | 1.13e-06 | 0,00178 |
| LEAP2 | NM_213788.1 | 56,1292973 | 33,2320901 | 22,8972072 | 1.71e-06 | 0,00248 |
| HAL | XM_001925061.1 | 52,4815207 | 30,3881709 | 22,0933498 | 2.6e-06 | 0,00323 |
| NNMT | NM_001123146.1 | 50,160218 | 28,6945508 | 21,4656673 | 3.6e-06 | 0,00391 |
| BTG3 | NM_001097517.1 | 43,2619928 | 25,2810348 | 17,9809581 | 2.23e-05 | 0,0168 |
| KRT18 | XM_003126180.1 | 35,7533516 | 17,804555 | 17,9487966 | 2.27e-05 | 0,0168 |
| CDKN1A | XM_001929558.1 | 61,9691073 | 44,7786882 | 17,1904191 | 3.38e-05 | 0,0218 |
| TSKU | XM_003129674.1 | 56,9571834 | 40,6969503 | 16,2602331 | 5.52e-05 | 0,0331 |
| FMO5 | XM_001928594.1 | 34,5734683 | 15,7248704 | 18,8485979 | 1.42e-05 | 0,0123 |
| TSKU | XM_003129672.1 | 59,9838628 | 42,5294505 | 17,4544123 | 2.94e-05 | 0,0198 |
| TSKU | XM_003129673.1 | 60,3171241 | 42,8759867 | 17,4411374 | 2.96e-05 | 0,0198 |
| CYP7A1 | NM_001005352.2 | 135,645781 | 114,76336 | 20,882421 | 4.88e-06 | 0,005 |
| HIST1H4K | XM_001928022.2 | 50,2590586 | 32,348052 | 17,9110066 | 2.31e-05 | 0,0168 |
| MBL2 | NM_214125.1 | 120,170016 | 77,5073935 | 42,6626223 | 6.5e-11 | 1.89e-07 |
| BCAM | XM_003127227.1 | 51,2186451 | 35,0938025 | 16,1248426 | 5.93e-05 | 0,0344 |
| HSD17B2 | NM_001167649.1 | 95,8572865 | 50,6868571 | 45,1704294 | 1.81e-11 | 6.28e-08 |
