# Supplementary material for: RNA Deep Sequencing Reveals Novel Candidate Genes and Polymorphisms in Boar Testis and Liver Tissues with Divergent Androstenone Levels
Source: PLoS One. 2013 May 16;8(5):e63259. doi: 10.1371/journal.pone.0063259 (PMC3655983; doi:10.1371/journal.pone.0063259)
Supplement: Table S2 — Sample read counts for polymorphisms on testis and liver DEGs. (DOC) [file pone.0063259.s003.doc]

**Table S2. Read counts for individual samples for identified polymorphisms in testis and liver tissues are give**

**Table S2.1: Sample read counts for polymorphisms on testis DEGs.**

**A1.DP : individual read depth for polymorphism in sample A1. Read depth value ‘NIL’ indicates that the polymorphism is identified in the sample group, but not in the sample. Read depth value ‘0’ indicates that polymorphism is not at all present in the sample group. A1.DP-A5.DP read depths for low androstenone testis sample. A6.DP-A10.DP read depths for high androstenone testis sample.**

| Refseq Id | Gene name | Chr | position | A1.DP | A2.DP | A3.DP | A4.DP | A5.DP | A6.DP | A7.DP | A8.DP | A9.DP | A10.DP |
| --- | --- | --- | --- | --- | --- | --- | --- | --- | --- | --- | --- | --- | --- |
| NM_213817 | IRG6 | 3 | 118838598 | NIL | NIL | 6 | 2 | NIL | 0 | 0 | 0 | 0 | 0 |
| XM_003124689 | HBA2 | 3 | 35253219 | 5 | 8 | 9 | 18 | 11 | 5 | 5 | 5 | NIL | 3 |
| XM_003124689 | HBA2 | 3 | 35253521 | NIL | 9 | 9 | 11 | 11 | 8 | 6 | 8 | NIL | 6 |
| XM_003124870 | LOC100516362 | 3 | 48107044 | 0 | 0 | 0 | 0 | 0 | 1 | 3 | 3 | NIL | NIL |
| XM_001928325 | CD244 | 4 | 93149337 | 0 | 0 | 0 | 0 | 0 | 4 | 21 | 1 | 12 | 3 |
| XM_003128168 | DSP | 7 | 4940734 | NIL | 2 | 1 | NIL | NIL | 0 | 0 | 0 | 0 | 0 |
| XM_003128168 | DSP | 7 | 4944881 | NIL | 6 | 1 | 2 | 1 | 0 | 0 | 0 | 0 | 0 |
| XM_001928679 | ARG2 | 7 | 99786827 | 0 | 0 | 0 | 0 | 0 | 2 | 1 | 4 | 8 | 3 |
| NM_214061 | MX1 | 13 | 144402807 | NIL | NIL | 18 | 5 | 5 | 0 | 0 | 0 | 0 | 0 |
| NM_214061 | MX1 | 13 | 144420441 | 0 | 0 | 0 | 0 | 0 | 3 | 4 | 11 | 3 | 4 |
| XM_001928671 | IFIT2 | 14 | 106102335 | NIL | NIL | 12 | 4 | 3 | 0 | 0 | 0 | 0 | 0 |
| XM_001928671 | IFIT2 | 14 | 106102694 | NIL | NIL | 5 | NIL | 2 | 0 | 0 | 0 | 0 | 0 |

**Table S2.2: Sample read counts for polymorphisms on liver DEGs.**

**B1.DP : individual read depth for polymorphism in sample B1. Read depth value ‘NIL’ indicates that the polymorphism is identified in the sample group, but not in the sample. Read depth value ‘0’ indicates that polymorphism is not at all present in the sample group. B1.DP-B5.DP read depths for low androstenone liver sample. B6.DP-B10.DP read depths for high androstenone liver sample**

| Refseq Id | Gene name | Chr | position | B1.DP | B2.DP | B3.DP | B4.DP | B5.DP | B6.DP | B7.DP | B8.DP | B9.DP | B10.DP |
| --- | --- | --- | --- | --- | --- | --- | --- | --- | --- | --- | --- | --- | --- |
| XM_001928594 | FMO5 | 4 | 104473018 | 15 | 26 | 6 | 5 | 5 | 0 | 0 | 0 | 0 | 0 |
| NM_001005352 | CYP7A1 | 4 | 77195279 | 2 | 48 | NIL | NIL | NIL | 0 | 0 | 0 | 0 | 0 |
| NM_001005352 | CYP7A1 | 4 | 77195397 | 6 | 140 | 1 | 1 | 6 | 7 | NIL | NIL | 15 | NIL |
| NM_001005352 | CYP7A1 | 4 | 77197364 | 7 | 50 | 1 | 2 | 2 | 6 | NIL | 1 | 9 | 1 |
| NM_001005352 | CYP7A1 | 4 | 77199510 | 1 | 13 | NIL | NIL | NIL | 0 | 0 | 0 | 0 | 0 |
| NM_001005352 | CYP7A1 | 4 | 77199576 | NIL | 11 | NIL | NIL | NIL | 0 | 0 | 0 | 0 | 0 |
| NM_001005352 | CYP7A1 | 4 | 77200294 | NIL | 11 | NIL | NIL | NIL | 0 | 0 | 0 | 0 | 0 |
| NM_001005352 | CYP7A1 | 4 | 77200408 | 1 | 14 | NIL | NIL | 3 | 0 | 0 | 0 | 0 | 0 |
| NM_001005352 | CYP7A1 | 4 | 77201533 | NIL | 76 | NIL | 1 | 5 | 0 | 0 | 0 | 0 | 0 |
| NM_001159615 | KRT8 | 5 | 16715238 | 29 | 18 | 47 | 37 | 39 | 77 | 52 | 174 | 6 | 28 |
| NM_001159615 | KRT8 | 5 | 16718099 | 26 | 10 | 42 | 31 | 36 | 61 | 23 | 117 | 4 | 21 |
| NM_001159615 | KRT8 | 5 | 16720725 | 5 | 7 | 14 | 12 | 8 | 23 | 7 | 36 | 2 | 9 |
| NM_001159615 | KRT8 | 5 | 16721108 | 6 | 4 | 10 | 6 | 5 | 14 | 7 | 22 | NIL | 10 |
| NM_001159615 | KRT8 | 5 | 16721708 | 48 | 45 | 53 | 52 | 38 | 65 | 39 | 74 | 17 | 55 |
| NM_001159615 | KRT8 | 5 | 16721831 | 124 | 116 | 136 | 127 | 91 | 173 | 95 | 179 | 31 | 152 |
| XM_003126180 | KRT18 | 5 | 16788495 | 52 | 32 | 30 | 27 | 7 | 0 | 0 | 0 | 0 | 0 |
| XM_003126180 | KRT18 | 5 | 16789240 | 10 | 6 | 3 | 4 | NIL | 16 | 4 | 15 | 3 | 7 |
| XM_003126180 | KRT18 | 5 | 16789379 | 60 | 40 | 34 | 32 | 2 | 85 | 21 | 89 | 17 | 70 |
| XM_003126180 | KRT18 | 5 | 16789412 | 69 | 48 | 34 | 32 | 2 | 102 | 21 | 102 | 17 | 70 |
| XM_003126180 | KRT18 | 5 | 16789808 | 147 | 120 | 83 | 70 | 22 | 167 | 51 | 166 | 64 | 159 |
| XM_003126180 | KRT18 | 5 | 16789954 | 0 | 0 | 0 | 0 | 0 | 17 | 2 | 22 | 3 | 9 |
| XM_001925061 | HAL | 5 | 82556747 | 0 | 0 | 0 | 0 | 0 | 2 | NIL | 11 | NIL | NIL |
| XM_001928022 | HIST1H4K | 7 | 22186329 | NIL | 28 | NIL | NIL | NIL | 0 | 0 | 0 | 0 | 0 |
| XM_001929558 | CDKN1A | 7 | 36992673 | 0 | 0 | 0 | 0 | 0 | 5 | 6 | 93 | 1 | 2 |
| XM_001929558 | CDKN1A | 7 | 36992792 | 1 | 2 | 4 | 3 | 3 | 3 | 3 | 57 | NIL | 1 |
| XM_003129674 | TSKU | 9 | 10759263 | 11 | 10 | 2 | NIL | NIL | 0 | 0 | 0 | 0 | 0 |
| NM_001123146 | NNMT | 9 | 40584781 | 10 | 3 | 7 | 5 | NIL | 14 | 9 | 18 | NIL | 3 |
| NM_214125 | MBL2 | 14 | 101464163 | 24 | 17 | 9 | NIL | 4 | NIL | 1 | 15 | NIL | NIL |
| NM_214125 | MBL2 | 14 | 101464174 | 42 | 26 | 12 | NIL | 4 | 1 | 2 | 17 | NIL | NIL |
| NM_214125 | MBL2 | 14 | 101464216 | 100 | 58 | 35 | 2 | 4 | 5 | 2 | 49 | 3 | 3 |
| NM_214125 | MBL2 | 14 | 101464268 | 112 | 53 | 42 | 5 | 3 | 3 | 1 | 48 | 3 | 5 |
| NM_214125 | MBL2 | 14 | 101464309 | 67 | 23 | 23 | 3 | 3 | NIL | 1 | 22 | NIL | 2 |
| NM_214125 | MBL2 | 14 | 101464842 | 34 | 19 | 8 | 2 | 1 | NIL | 2 | 9 | 1 | 1 |
| NM_214125 | MBL2 | 14 | 101467788 | 188 | 68 | 75 | 5 | 3 | 7 | 3 | 76 | 7 | 3 |
| XM_001928302 | SDS | 14 | 38865735 | 0 | 0 | 0 | 0 | 0 | 4 | NIL | 9 | NIL | 1 |
| XM_001928302 | SDS | 14 | 38868514 | 7 | 14 | 9 | 14 | 14 | 0 | 0 | 0 | 0 | 0 |
