# Supplementary material for: RNA Deep Sequencing Reveals Novel Candidate Genes and Polymorphisms in Boar Testis and Liver Tissues with Divergent Androstenone Levels
Source: PLoS One. 2013 May 16;8(5):e63259. doi: 10.1371/journal.pone.0063259 (PMC3655983; doi:10.1371/journal.pone.0063259)
Supplement: Table S3 — Selected SNP detected by RNA-seq that were validated using RFLP. (DOC) [file pone.0063259.s004.doc]

Supplementary Table S3. Selected SNP detected by RNA-seq that were validated using RFLP

| Gene | Chromosome | SNP | Position | Frequency  (%) | Confirmed* |
| --- | --- | --- | --- | --- | --- |
| IRG6 | 3 | G/A | 118838598 | 15/85 | Yes |
| DSP | 7 | C/T | 4944881 | 49/51 | Yes |
| MX1 | 13 | C/T | 144420441 | 69/31 | Yes |
| IFIT2 | 14 | G/T | 106102335 | 24/76 | Yes |
| FMO5 | 4 | G/A | 104473018 | 47/53 | Yes |
| CYP7A1 | 4 | A/G | 77201533 | 10/90 | Yes |
| KRT18 | 5 | G/A | 16788495 | 89/11 | Yes |

*SNP confirmed by RFLP genotyping of DNA
