# Supplementary material for: RNA Deep Sequencing Reveals Novel Candidate Genes and Polymorphisms in Boar Testis and Liver Tissues with Divergent Androstenone Levels
Source: PLoS One. 2013 May 16;8(5):e63259. doi: 10.1371/journal.pone.0063259 (PMC3655983; doi:10.1371/journal.pone.0063259)
Supplement: Table S4 — Genotype, allele frequencies and the chi-square test of selected SNPs validated using RFLP. (DOC) [file pone.0063259.s005.doc]

Supplementary Table S4. Genotype, allele frequencies and the chi-square test of selected SNPs validated using RFLP

| Polymorphism position | Number of boars | Genotype frequency | | |  | Allele frequency | | p-value | Chi-square test |
| --- | --- | --- | --- | --- | --- | --- | --- | --- | --- |
|  |  | GG | GA | AA |  | G | A |  | χ^2^ |
| IRG6 g.118838598G>A | 100 | 0.06(6) | 0.17(17) | 0.77(77) |  | 0.15 | 0.85 | 0.009 | 0.10 |
|  |  | CC | CT | TT |  | C | T |  |  |
| DSP g.4944881 C>T | 100 | 0.26(26) | 0.45 (45) | 0.29(29) |  | 0.49 | 0.51 | 0.191 | 0.01 |
|  |  | CC(n) | CT(n) | TT(n) |  | A | G |  |  |
| MX1 g.144402807 C>T | 100 | 0.35(35) | 0.52 (51) | 0.14(13) |  | 0.69 | 0.31 | 0.01 | 0.01 |
|  |  | GG | GT | TT |  | G | T |  |  |
| IFIT2 g.106102335 G>T | 100 | 0.16(16) | 0.16(16) | 0.68(68) |  | 0.24 | 0.76 | 0.004 | 0.32 |
|  |  | GG | GA | AA |  | G | A |  |  |
| FMO5 g.104473018 G>A | 100 | 0.08 (8) | 0.77(77) | 0.15(15) |  | 47 | 53 | 0.04 | 0.30 |
|  |  | AA | AG | GG |  | A | G |  |  |
| CYP7A1 g.77201533 A>G | 100 | 0.05(5) | 0.10(10) | 0.85(85) |  | 0.10 | 0.90 | 0.004 | 0.20 |
|  |  | GG | GA | AA |  | G | A |  |  |
| KRT18 g.16788495 G>A | 100 | 0.85(85) | 0.09(9) | 0.06(6) |  | 0.89 | 0.11 | 0.001 | 0.27 |
